# Seroprevalence of SARS-Cov-2 Antibodies in Adults, Arkhangelsk, Russia

**DOI:** 10.3201/eid2802.211640

**Published:** 2022-02

**Authors:** Ekaterina Krieger, Alexander Kudryavtsev, Ekaterina Sharashova, Vitaly Postoev, Natalia Belova, Leonid Shagrov, Julia Zvedina, Oxana Drapkina, Anna Kontsevaya, Svetlana Shalnova, Tormod Brenn, Vladimir M. Shkolnikov, Rosalind M. Eggo, David A. Leon

**Affiliations:** UiT The Arctic University of Norway, Tromso, Norway (E. Krieger, A. Kudryavtsev, E. Sharashova, T. Brenn, D.A. Leon);; Northern State Medical University of the Ministry of Health of the Russian Federation, Arkhangelsk, Russia (E. Krieger, A. Kudryavtsev, V. Postoev, N. Belova, L. Shagrov, J. Zvedina);; National Medical Research Centre for Therapy and Preventive Medicine, Moscow, Russia (O. Drapkina, A. Kontsev, S. Shalnova);; Max-Planck-Institute for Demographic Research, Mecklenburg, Germany (V.M. Shkolnikov);; National Research University Higher School of Economics, Moscow (V.M. Shkolnikov, D.A. Leon);; London School of Hygiene & Tropical Medicine, London, UK (R.M. Eggo, D.A. Leon)

**Keywords:** COVID-19, SARS-CoV-2, severe acute respiratory syndrome coronavirus 2, viruses, respiratory infections, zoonoses, seroprevalence, Russia, epidemiology, antibodies, *Suggested citation for this article*: Krieger E, Kudryavtsev A, Sharashova E, Postoev V, Belova N, Shagrov L, et al. Seroprevalence of SARS-CoV-2 antibodies in adults, Arkhangelsk, Russia. Emerg Infect Dis. 2022 Feb [*date cited*]. https://doi.org/10.3201/eid2802.210403

## Abstract

Population-based data on coronavirus disease in Russia and on the immunogenicity of the Sputnik V vaccine are sparse. In a survey of 1,080 residents of Arkhangelsk 40–75 years of age, 65% were seropositive for IgG. Fifteen percent of participants had been vaccinated; of those, 97% were seropositive.

Russia is one of the few countries to have produced a coronavirus (COVID-19) vaccine (*1*). It has also experienced substantial excess deaths during the pandemic (*2*). Few published estimates of antibody seroprevalence for severe acute respiratory syndrome coronavirus 2 (SARS-CoV-2) in Russia exist. A St. Petersburg survey in June 2020 used random-digit dialing to contact 66,250 residents; of those, 1,038 provided a blood sample, and the samples had 9%–10% seropositivity (*3*). A study conducted in Chelyabinsk (September 28–December 30, 2020) recruited 1,091 high-risk workers (healthcare workers, education staff, and supermarket employees) >18 years of age. Of the 882 screened, 25% were seropositive for IgG (*4*). We are not aware of any seroprevalence estimates from Russia based on samples collected in 2021 that have appeared in the scientific literature. 

We interviewed and obtained blood samples from 1,080 adults 40–75 years of age who were residents of the city of Arkhangelsk in northwest Russia during February 24–May 28, 2021. We obtained participants for this study from 2,258 invitations sent to persons who had taken part in the Know Your Heart study (*5*) (2015–2018), which was based on a random sample of the city population (Appendix). The ethics committee of the Northern State Medical University approved our study proposal and protocol on February 17, 2021.

We used a Vector Best ELISA assay (D-5501 SARS-CoV-2-IgG-EIA-BEST; https://vector-best.ru) to analyze qualitatively detected IgG directed against SARS-CoV-2 in human blood serum samples. Data are limited on the performance of this immunoassay, in particular, on its sensitivity for infections that occurred >3 weeks previously. According to the manufacturer, the assay has a sensitivity of 72% when performed 6–12 days after infection and ≈100% at 13–20 days (*6*). An independent assessment of the Vector Best ELISA assay found a sensitivity of 89% and a specificity of 100%, derived from comparisons of test results in prepandemic samples (negative controls) and PCR positive samples for SARS-CoV-2 (*7*). We estimated seroprevalence adjusted for test performance (89% sensitivity, 100% specificity) using the equation (crude prevalence + test specificity − 1)/(sensitivity + specificity − 1) (*8*). We calculated 95% CIs for the adjusted estimates of seroprevalence using the R package bootComb (https://www.r-project.org).

Of the 1,080 samples (634 women, mean age 55 years), we excluded 13 who had an equivocal test result from analysis. Of the 1,067 remaining samples, 690 (65%) were seropositive for IgG (Table). Seroprevalence adjusted for test characteristics was 72.6% (95% CI 64.2%–83.1%).

**Table:** Seroprevalence of severe acute respiratory syndrome coronavirus 2 in adults, Arkhangelsk, Russia

Characteristic	Unvaccinated			Vaccinated*			Total	
	No. seropositive/total (%)	Adjusted seroprevalence, % (95% CI)†		No. seropositive/total (%)	Adjusted seroprevalence, % (95% CI)†		No. seropositive/total (%)	Adjusted seroprevalence, % (95% CI)†
Sex								
F	332/553 (60)	67.4 (58.4–77.9)		72/81 (89)	99.7 (87.1–99.9)		404/634 (64)	71.5 (62.6–82.3)
M	208/352 (59)	66.3 (56.5–77.3)		78/81 (96)	100 (93.2–100)		286/433 (66)	74.1 (64.5–85.6)
Age, y								
40–54	291/461 (63)	70.8 (61.4;81.8)		35/38 (92)	100 (84.8–100)		326/499 (65)	73.3 (64.0–84.6)
55–64	181/317 (57)	64.1 (54.1–75.0)		38/43 (88)	99.1 (82.6–100)		219/360 (61)	68.3 (58.4–79.4)
>65	68/127 (54)	60.1 (46.9–73.1)		77/81 (95)	100 (92.4–100)		145/208 (70)	78.2 (67.0–91.2)
Education								
Secondary and lower	26/47 (55)	62.1 (42.7–81.0)		9/9 (100)	100 (66.7–100)		35/56 (63)	70.1 (52.5–88.1)
Specialized secondary	253/433 (58)	65.6 (56.1–76.0)		81/87 (93)	100 (91.2–100)		334/520 (64)	72.1 (62.9–83.2)
Higher	261/425 (61)	68.9 (59.3–79.8)		60/66 (91)	100 (88.0–100)		321/491 (65)	73.3 (64.0–84.6)
Week of test								
7–14	395/651 (61)	68.1 (59.3–78,4)		49/58 (84)	94.8 (81.0–100)		444/709 (63)	70.3 (61.6–80.8)
15–21	145/254 (57)	64.0 (53.4–75.3)		101/104 (97)	100 (94.8–100)		246/358 (69)	77.1 (67.1–89.1)
Self-reported prior symptoms of infection								
No	172/477 (36)	40.5 (31.7–47.8)		133/143 (93)	100 (92.9–100)		305/620 (49)	55.2 (46.6–64.0)
Yes	248/256 (97)	100 (96.9–100)		8/9 (89)	99.7 (56.8–100)		256/265 (97)	100 (96.7–100)
Do not know	120/172 (70)	78.3 (66.5–91.6)		9/10 (90)	100 (60.4–100)		129/182 (71)	79.5 (68.1–92.8)
Total	540/905 (60)	66.9 (58.6–76.9)		150/162 (93)	100 (92.9–100)		690/1067 (65)	72.6 (64.2–83.1)

*Received >1 dose. †Values >100% were rounded to 100%. ‡Weeks 7–14 are February 24–April 11 and weeks 15–21 are April 12–May 28, 2021.

Seroprevalence did not substantively differ by sex or by educational level. Of the 162 participants (15%) who reported having been vaccinated. 150 (93%) were seropositive. Among the 31 who received 1 dose, 20 (65%) were seropositive; of the 131 who had received 2 doses, 130 (99%) were seropositive. Of the 905 participants who said they had not been vaccinated, 256 said that they had previously been ill with COVID-19; of those, 248 (97%) were seropositive. Of those who stated they had not been vaccinated and did not report having previously been ill with COVID-19, 292 (45%) were seropositive, suggesting an appreciable level of unrecognized infection. Our overall estimates of seroprevalence (crude 65%, adjusted 72.6%) is appreciably higher than found in St Petersburg in June 2020 (*3*) (10%) or in Chelyabinsk (25%) in September–December 2020 (*4*). This result is consistent with the second wave of the pandemic in Russia (peak November–December 2020) being larger than the first (peak May–June 2020); our study started during the vaccination period.

Deployment of COVID-19 vaccine, mostly Sputnik V, in the Arkhangelsk region started in mid-January 2021; 11% of the population received >1 dose by May 30, 2021 (*9*). Our study covered an urban sample from the city of Arkhangelsk, the capital of the region. Our estimate of 15% coverage of the study population may be higher because the regional estimates included data from more dispersed communities in. Nevertheless, our vaccination rates were low compared with rates in most European Union and European Economic Area countries as reported in June 2021 by the European Centre for Disease Prevention and Control (*10*). Given the vaccination rate in the sample was 15% but the antibodies were present in 65% of participants, we suspect that most of the seropositive results were the result of acquired infection.

Russia is geographically the largest country in the world; its regions vary considerably in terms of socioeconomic level, climate, and healthcare provision. Our study results are restricted to an adult population and cannot be generalized to the total population of Arkhangelsk region or to Russia. The high levels of seroprevalence among vaccinated participants confirms the immunogenicity of the Sputnik vaccine and suggests that it can protect the population if the proportion vaccinated is increased substantially. We recommend further population-based seroprevalence studies, using World Health Organization–approved tests, for public health efforts in the COVID-19 pandemic.

Appendix Additional information from a study of seroprevalence of SARS-Cov-2 antibodies in adults, Russia.

## References

[1] Logunov DY, Dolzhikova IV, Zubkova OV, Tukhvatullin AI, Shcheblyakov DV, Dzharullaeva AS, et al. Safety and immunogenicity of an rAd26 and rAd5 vector-based heterologous prime-boost COVID-19 vaccine in two formulations: two open, non-randomised phase 1/2 studies from Russia. Lancet. 2020;396:887–97. 10.1016/S0140-6736(20)31866-3 32896291 PMC7471804

[2] Karlinsky A, Kobak D. Tracking excess mortality across countries during the COVID-19 pandemic with the World Mortality Dataset. eLife. 2021;10:10. 10.7554/eLife.69336 34190045 PMC8331176

[3] Barchuk A, Skougarevskiy D, Titaev K, Shirokov D, Raskina Y, Novkunkskaya A, et al. Seroprevalence of SARS-CoV-2 antibodies in Saint Petersburg, Russia: a population-based study. Sci Rep. 2021;11:12930. 10.1038/s41598-021-92206-y 34155259 PMC8217236

[4] Zurochka A, Dobrinina M, Zurochka V, Hu D, Solovyev A, Ryabova L, et al. Seroprevalence of SARS-CoV-2 antibodies in symptomatic individuals is higher than in persons who are at increased risk exposure: the results of the single-center, prospective, cross-sectional study. Vaccines (Basel). 2021;9:627. 10.3390/vaccines9060627 34207919 PMC8229032

[5] Cook S, Malyutina S, Kudryavtsev AV, Averina M, Bobrova N, Boytsov S, et al. Know Your Heart: Rationale, design and conduct of a cross-sectional study of cardiovascular structure, function and risk factors in 4500 men and women aged 35-69 years from two Russian cities, 2015-18. Wellcome Open Res. 2018;3:67. 10.12688/wellcomeopenres.14619.1 30123849 PMC6073094

[6] Kuvshinova IN, Nekrasov BG, Livitskaya NI, Molodykh SV, Rukavishnikov M. Sensitivity and specificity of reagent kits of JSC “Vector-Best” for the detection of immunoglobulins of different classes to SARS-CoV-2 [in Russian]. Spravochnik Zaveduyushchego KDL. 2021;10:27–32.

[7] Barchuk A, Shirokov D, Sergeeva M, Tursun Zade R, Dudkina O, Tychkova V, et al. Evaluation of the performance of SARS—CoV—2 antibody assays for a longitudinal population-based study of COVID—19 spread in St. Petersburg, Russia. J Med Virol. 2021;93:5846–52. 10.1002/jmv.27126 34081328 PMC8242745

[8] Sempos CT, Tian L. Adjusting coronavirus prevalence estimates for laboratory test kit error. Am J Epidemiol. 2021;190:109–15. 10.1093/aje/kwaa174 32803245 PMC7454308

[9] Government of Russia. The number of people vaccinated against coronavirus in Arkhangelsk [in Russian]. 2021 [cited 2021 Sep 20]. https://gogov.ru/covid-v-stats/arkhangelsk

[10] European Centre for Disease Prevention and Control. Overview of the implementation of COVID-19 vaccination strategies and deployment plans in the EU/EEA, 14 June 2021. 2021 [cited 2021 Sep 20]. https://www.ecdc.europa.eu/en/publications-data/overview-implementation-covid-19-vaccination-strategies-and-deployment-plans

