# Supplementary material for: Seroprevalence of SARS-Cov-2 Antibodies in Adults, Arkhangelsk, Russia
Source: Emerg Infect Dis. 2022 Feb;28(2):463–5. doi: 10.3201/eid2802.211640 (PMC8798687; doi:10.3201/eid2802.211640)
Supplement: Appendix — Additional information from a study of seroprevalence of SARS-Cov-2 antibodies in adults, Russia. [file 21-1640-Techapp-s1.pdf]

Article DOI: https://doi.org/10.3201/eid2802.211640

Seroprevalence of SARS-Cov-2 Antibodies in Adults, Arkhangelsk, Russia

Appendix

A potential limitation of this study is that the population we studied may not be fully representative of the target population of citizens of Arkhangelsk of the same age. This has 2 components. First, the sampling frame for the seroprevalence study was from the previous Know Your Heart (KYH) study conducted 2015–2018. KYH was itself based on a random sample of all persons 35–69 years of age residing in the city of Arkhangelsk. Although the response rate for this initial study was 53%, the educational profile of those who were recruited to the study was very similar to that expected on the basis of 2010 Russian Census data for the city (*1*). This finding, together with emerging evidence that response rates may not be as strongly related to nonresponse bias (*2*), suggests that although we sampled a particular age-range, the sampling frame is probably representative of the population of the city of Arkhangelsk.

The second issue of representativeness concerns the extent to which those participants in the recent seroprevalence study are similar in key respects to the sampling frame from the KYH study. Of the 2,380 KYH participants, we excluded 122 persons from consideration for the following reasons: 56 indicated at the KYH survey that they did not wish to be contacted to take part in further research, 61 had died before the study inception date, and 5 were ≥75 years of age. Overall, 2,258 people were invited to take part in 2021; a total of 1,080 (47.8%) provided blood samples for assessing seroprevalence.

We have compared the similarity of the 1,080 participants in the seroprevalence survey to the 2,380 persons in the sampling frame (Appendix Table). The sex and age distributions were very similar. However, the proportion of participants with higher education in the 2021 seroprevalence study was larger than in the sampling frame. However, we did not observe an association between education and seroprevalence, at least among the responders.

Our study had several limitations. Samples were taken over 4 months during February 24–May 28, 2021, because the fieldwork was nested within a much larger national multicenter

survey of the prevalence of risk factors of cardiovascular diseases in Russia (study ESSE-RF-3). Participants of this study underwent extensive tests as part of the ESSE-RF-3 protocol. Capacity limitations meant that we could invite a maximum of 25 participants per day. We noted, however, that the ESSE-RF study itself aimed to get a representative sample of the population and was in no way restricted to those who had cardiovascular problems. Although we collected the samples over an extended period during which the infection rates changed, we regard our estimate as an average of positivity over the period studied. Nevertheless, we could underestimate the seroprevalence due to low sensitivity in the 12 days following infection or sensitivity waning with increased time from the disease onset. Finally, a limitation of our findings was the small sample sizes we used for some of our analyses.

References

1. Cook S, Malyutina S, Kudryavtsev AV, Averina M, Bobrova N, Boytsov S, et al. Know Your Heart: rationale, design and conduct of a cross-sectional study of cardiovascular structure, function and risk factors in 4,500 men and women aged 35–69 years from two Russian cities, 2015-18 [version 2–referees: 3 approved]. Wellcome Open Research 2018;3 https://doi.org/10.12688/wellcomeopenres.14619.1

2. Hendra R, Hill A. Rethinking response rates: new evidence of little relationship between survey response rates and nonresponse bias. Eval Rev. 2019;43:307–30. https://doi.org/10.1177/0193841X18807719

Appendix Table. Comparisons of participants in study of seroprevalence of severe acute respiratory syndrome coronavirus 2 antibodies, Russia

Characteristic	Resurvey sample, N=1,067 (%)	Know Your Heart study, N=2,380 (%)	p value
Sex*			
F	629 (59.0)	1,377 (58.3)	0.72
M	438 (41.0)	985 (41.7)	
Median age at baseline, quartiles Q1 and Q3	51 (Q_1=44–Q_3=59)	54 (Q_1=45–Q_3=62)	<0.01†
Education*			<0.01
Secondary and lower	56 (5.2)	174 (7.4)	
Specialized secondary	520 (48.7)	1,269 (53.7)	
Higher	491 (46.0)	919 (38.9)	

*Determined by χ^2 test.
†Determined by Mann–Whitney U-test.
